# Exogenous Short Chain Fatty Acid Effects in APP/PS1 Mice

**DOI:** 10.3389/fnins.2022.873549

**Published:** 2022-07-04

**Authors:** Diana J. Zajac, Benjamin C. Shaw, David J. Braun, Stefan J. Green, Joshua M. Morganti, Steven Estus

**Affiliations:** ^1^Department of Physiology and Sanders-Brown Center on Aging, University of Kentucky, Lexington, KY, United States; ^2^Department of Neuroscience and Sanders-Brown Center on Aging, University of Kentucky, Lexington, KY, United States; ^3^Genome Research Core, Research Resources Center, University of Illinois at Chicago, Chicago, IL, United States

**Keywords:** SCFAs, Alzheimer’s, microbiome, amyloid, behavior

## Abstract

Elucidating the impact of the gut microbiome on Alzheimer’s Disease (AD) is an area of intense interest. Short chain fatty acids (SCFAs) are major microbiota metabolites that have been implicated as a mediator of gut microbiome effects in the brain. Here, we tested the effects of SCFA-treated water vs. saline-treated water on APPswe/PSEN1dE9 mice maintained under standard laboratory conditions. Mice were treated with SCFAs from five months of age until ten months of age, when they were evaluated for microbiome profile, impaired spatial memory as evaluated with the radial arm water maze, astrocyte activation as measured by Gfap expression and amyloid burden as assessed by histochemistry and MSD ELISA. We report that SCFA treatment increased alpha-diversity and impacted the gut microbiome profile by increasing, in part, the relative abundance of several bacteria that typically produce SCFAs. However, SCFA treatment did not significantly affect behavior. Similarly, SCFAs did not affect cortical or hippocampal astrocyte activation observed in the APP/PS1 mice. Lastly, although robust levels of soluble and insoluble amyloid were present in the APP/PS1 mice, SCFA treatment had no effect on these indices. Overall, our findings are that SCFA treatment modifies the microbiome in a fashion that may increase further SCFA production. However, SCFA treatment did not alter behavior, astrocyte activation, nor amyloid neuropathology in APP/PS1 mice maintained with a conventional microbiome.

## Introduction

The impact of the gut microbiome on Alzheimer’s Disease (AD) is an area of intense current scrutiny [reviewed in Seo et al. (2019); Sorboni et al. (2022)]. Several studies have suggested differences in the gut microbiome between AD and non-AD individuals (Cattaneo et al., 2017; Vogt et al., 2017; Zhuang et al., 2018). Whether the relationship between the gut microbiome and AD risk extends from correlation to causality is unclear, although several reports have found that Aβ burden in murine models is reduced in gnotobiotic mice or mice treated with antibiotics (Minter et al., 2016, 2017; Bonfili et al., 2017; Harach et al., 2017; Colombo et al., 2021; Guilherme et al., 2021). Hence, the gut microbiome may emerge as a modulator of AD risk.

Short chain fatty acids are a major microbiota metabolite that have been suggested to mediate gut microbiome effects in the brain [reviewed in Erny et al. (2017); Martin-Gallausiaux et al. (2021)]. The major source of SCFAs in the body is microbial digestion of resistant starch. Recently, SCFA treatment was reported to increase amyloid burden in specific-pathogen-free (SPF) APP/PS1 mice (Colombo et al., 2021) while butyrate treatment was reported to decrease amyloid burden in SPF 5xFAD mice (Jiang et al., 2021) and in 5xFAD mice maintained on a conventional microbiome (Fernando et al., 2020). We refer to a conventional microbiome as the microbiome of mice that were conventionally raised, as opposed to germ free or SPF mice, which have laboratory controlled/limited microbiomes. Here, we tested the effects of SCFAs on mice maintained under standard laboratory conditions with a conventional microbiome. For this effort, five-month-old APP/PS1 mice were treated with SCFAs until ten months of age, and then evaluated for microbiome profile, spatial memory deficit, glial activation, and amyloid burden. We report that SCFA treatment impacted the gut microbiome but not memory impairment, glial activation, or amyloid burden in this paradigm.

## Methods

### Animals

APP/PS1 (APPswe, PSEN1dE9) are double transgenic mice expressing a chimeric mouse/human amyloid precursor protein (Mo/HuAPP695swe) and a human *PSEN1* gene lacking exon 9 (PS1-dE9) (Jankowsky et al., 2004). We chose this mouse model because the mice begin to develop Aβ deposits by six months of age, with abundant plaques in the hippocampus and cortex by 9 months (Jankowsky et al., 2004). Plaques continue to increase up to around 12 months of age (Garcia-Alloza et al., 2006). This is a less aggressive amyloid phenotype with a delayed onset compared to other mouse models, such as the 5xFAD (Webster et al., 2014), and we hypothesized that a mild agent, such as SCFA treatment, would be more likely to have an effect in the APP/PS1 model. Behavioral deficits have been reported across cognitive domains, although severity and timing depend on the specific behavioral tests (Janus et al., 2015). Mice were bred by crossing *APP/PS1* carriers with wild-type C57Bl/6J mice. At weaning, mice were separated by sex and housed as mixed genotypes with 2–5 mice per cage. Non-APP/PS1 (WT) littermates served as control mice.

Mice were maintained on standard mouse chow (Teklad Global 18% Protein Rodent Diet) in individually ventilated cages. This diet consists of ground wheat, ground corn, wheat middlings, dehulled soybean meal, corn gluten meal, soybean oil, and brewers dried yeast, as well as vitamins and minerals (Envigo, 2022). Soluble starches such as inulin that contribute to SCFA production are present in these ingredients, although the specific amounts are not available. Another dietary component, brewers dried yeast, is also known to impact the gut microbiome (Nakashimada et al., 2011). Each of the mice in this study were maintained on the same chow for the duration of the study.

Beginning at five months of age, drinking water was supplemented with either SCFAs (67.5 mM sodium acetate, 25 mM sodium propionate, 40 mM sodium butyrate, pH 6.8) or with sodium chloride (132.5 mM) (Erny et al., 2015). This solution was administered *via* water bottles and was made fresh weekly for a total of five additional months. Equivalent amounts of water were consumed by each group. This approach has been previously used and shown to significantly increase plasma concentrations of acetate, propionate and butyrate in murine models, including an APPPS1 mouse model (Pomare et al., 1985; Colombo et al., 2021).

### Microbiome Analysis

Fecal samples were collected on the day of euthanasia. The number of mice included 34 males (16 APP/PS1 which included eight on SCFA and eight on saline, and 18 WT which included 11 on SCFA and seven on saline), and 32 females (14 APP/PS1 which included five on SCFA and nine on saline, and 18 WT which included 10 on SCFA and eight on saline). DNA was isolated by using a QIAamp PowerFecal Pro DNA Kit (QIAGEN). Genomic DNA was polymerase chain reaction (PCR) amplified with primers CS1_515F and CS2_806R (modified from the primer set employed by the Earth Microbiome Project (EMP; GTGYCAGCMGCCGCGGTAA and GGACTACNVGGGTWTCTAAT) targeting the V4 regions of microbial small subunit ribosomal RNA genes. Amplicons were generated using a two-stage PCR amplification protocol as described previously (Naqib et al., 2018). The primers contained 5′ common sequence tags (known as common sequence 1 and 2, CS1 and CS2). First stage PCR amplifications were performed in 10 ml reactions in 96-well plates, using MyTaq HS 2X mastermix (Bioline). PCR conditions were 95°C for 5 min, followed by 28 cycles of 95°C for 30 s, 55°C for 45 s and 72°C for 60 s.

Subsequently, a second PCR amplification was performed in 10 μl reactions in 96-well plates. A mastermix for the entire plate was made using MyTaq HS 2X mastermix. Each well received a separate primer pair with a unique 10-base barcode, obtained from the Access Array Barcode Library for Illumina (Fluidigm, South San Francisco, CA, United States; Item# 100-4876). Cycling conditions were: 95°C for 5 min, followed by 8 cycles of 95°C for 30 s, 60°C for 30 s and 72°C for 30 s. Samples were then pooled, purified, and sequenced on an Illumina MiniSeq platform employing paired-end 2 × 153 base reads. Fluidigm sequencing primers, targeting the CS1 and CS2 linker regions, were used to initiate sequencing. De-multiplexing of reads was performed on instrument. Library preparation, pooling, and sequencing were performed at the University of Illinois at Chicago Genome Research Core (GRC) within the Research Resources Center (RRC).

Forward and reverse reads were merged using PEAR (Zhang et al., 2014) and trimmed based on a quality threshold of *p* = 0.01. Ambiguous nucleotides and primer sequences were removed and sequences shorter than 225 bp were discarded. Chimeric sequences were identified and removed using the USEARCH algorithm with a comparison to the Silva 132_16S reference database (Edgar, 2010; Silva et al., 2016). Amplicon sequence variants (ASVs) were identified using DADA2 (Glockner et al., 2017) and their taxonomic annotations determined using the UCLUST algorithm and Silva 132_16S reference with a minimum similarity threshold of 90% (Edgar, 2010; Silva et al., 2016).

This sequencing effort yielded 4,443,016 reads. Raw sequence data files were submitted to the Sequence Read Archive (SRA) of the National Center for Biotechnology Information (NCBI) (BioProject #: PRJNA809693). One sample with fewer than 30,000 reads each was discarded. The average read count per sample was 67,318, where the minimum was 40,337 and the maximum was 134, 816. Using MicrobiomeAnalyst (Dhariwal et al., 2017) (updated version October 2021), samples were rarified to the minimum library size for each dataset. Low abundance ASVs were removed, i.e., ASVs with < three counts in > 90% of the samples were removed, and low variance ASVs were also removed, i.e., ASVs whose inter-quantile range was in the lowest 10% (Dhariwal et al., 2017). These corrections reduced the number of ASVs from 156 to 87 and 84 ASVs in the male and female mice datasets respectively. Count data were normalized with a centered log-ratio transformation.

Alpha-diversity was assessed using the Shannon H diversity index (Hammer et al., 2001) with statistical significance determined by Kruskal-Wallis tests. Beta-diversity was assessed using Principal Coordinates Analysis (PCoA) of Bray-Curtis matrices with statistical significance determined by Permutational Multivariate Analysis of Variance (PERMANOVA) (Oksanen et al., 2011). Taxa that associated with SCFA or *APP/PS1* status were determined using a classical univariate analysis with a Kruskal–Wallis test. A false discovery rate (FDR) approach was used to correct for multiple testing (Dhariwal et al., 2017).

Bacteria associated with SCFA treatment or *APP/PS1* status were identified by a linear discriminant analysis of effect size (LefSe) approach (Segata et al., 2011). Significance thresholds were set to 0.05 for the alpha values for Kruskal-Wallis/Wilcoxon tests and 2.0 for the logarithmic linear discriminant analysis (LDA) score, using a one-against-all multi-class analysis approach. These results were then plotted as a cladogram using the Huttenhower Galaxy resources to document the phylogenetic relatedness of SCFA associations with the bacteria at each taxonomic level (Segata et al., 2011).

### Behavioral Tests

Testing was performed by the Sanders-Brown Rodent Behavior Facility. Since robust behavioral deficits were previously identified with a Radial Arm Water Maze (RAWM) (Reiserer et al., 2007), we used this learning and memory task which takes advantage of the simple motivation provided by immersion into water. The radial arm water maze has been well characterized and used many times to detect a deficit in reference and working memory in the APP/PS1 mouse model (Park et al., 2006; Sood et al., 2007; Volianskis et al., 2010). The two-day RAWM test of spatial reference memory (Alamed et al., 2006) was performed as previously described (Webster et al., 2013; Bachstetter et al., 2015). Mice were trained to find a hidden platform in one of eight arms using extramaze visual cues and were scored for number of errors made before finding the platform. The platform was kept in the same goal arm for each mouse, with the start arm sequence randomized such that all mice started from each of the other five arms (not including the goal nor the two arms directly adjacent) three times per day. Each trial lasted until the mouse found the platform or 60 s had elapsed, whichever occurred first. Mice that failed to reach the platform in 60 s were gently guided there and allowed to remain for 15 s. Errors were counted as a mouse fully entering an incorrect (non-goal) arm or spending 15 consecutive seconds or longer in the same non-goal zone. On day one, mice were trained with 12 alternating hidden and visible platform trials followed by three hidden platform trials; averaged across three consecutive trials into five blocks. On day two, mice again underwent 15 trials but with a hidden platform only. To ensure that any observed effects were not due to differences in vision or swimming ability, each mouse was tested in an open pool with no obstacles and the platform clearly identified (Supplementary Figure 1). Each of the mice analyzed for microbiome were analyzed for behavior. Since females are known to have a larger amyloid burden in this model (Onos et al., 2019), results from males and females were analyzed separately by using a general linear model with treatment and transgene status and a treatment-transgene interaction term as main effects.

### Gfap Expression and Amyloid β (Aβ) Accumulation

A random subset of mice were analyzed further for Gfap expression and Aβ quantitation. The number of mice included 22 males (nine APP/PS1 which included five on SCFA and four on saline, and 13 WT which included six on SCFA and seven on saline), and 16 females (eight APP/PS1 which included four on SCFA and four on saline, and eight WT which included three on SCFA and five on saline). Mice were deeply anesthetized with 5% isoflurane and then underwent transcardial perfusion with 50 ml ice-cold phosphate-buffered saline (PBS) at a flow rate of 10 ml/min before decapitation and brain removal and dissection. The right hemisphere was post-fixed in 4% paraformaldehyde for 24 h at 4 °C and cryo-protected in 30% sucrose for at least 48 h at 4 °C. Brains were then embedded in a solid matrix at 40 per block and sectioned coronally (MultiBrain processing by NeuroScience Associates, Knoxville, TN, United States). For Gfap immunohistochemistry, free floating sections were treated with hydrogen peroxide, blocked and immunostained with Gfap (Dako, Catalog#: Z0334,1/1000), incubated overnight at room temperature, and labeled cells detected with a biotinylated secondary antibody (Vector Lab), and diaminobenzidine tetrahydrochloride (DAB). To visualize amyloid deposits, sections were then subjected to a Campbell–Switzer silver stain. A detailed protocol for this stain can be found online at the NeuroScience Associates website: http://www.neuroscienceassociates.com/Documents/Publications/campbell-switzer_protocol.htm.

Gfap and amyloid staining was quantified in the dorsal hippocampus and overlying cortex by manually outlining these regions of interest in the HALO analysis suite (Indica Labs, version 2.3.2089.34) by an investigator blinded to experimental groups. The algorithm minimum intensity settings for all analyses were manually thresholded based upon negative control. Cortical and hippocampal analyses of Gfap and amyloid staining was quantified by using the area quantification algorithm (Area Quantification v.2.2.1) applied to the traced region across three-to-four sections per animal to give a single average count per square millimeter of tissue per region. Results from males and females were analyzed separately by using a general linear model with treatment and transgene status and a treatment-transgene interaction term as main effects.

### MesoScale Discovery (MSD) Multiplex ELISA

Hippocampi and cortices were dissected from the left hemisphere to approximate the regions outlined for the amyloid staining analyses in the right hemisphere. Samples were snap frozen and kept at –80°C until Aβ quantitation. For this analysis, a random subset of APP/PS1 mice included nine males (which included four on SCFA and five on saline), and nine females (which included five on SCFA and four on saline). Soluble Aβ peptides were then quantified with an MSD approach as described previously (Braun et al., 2020). Briefly, the PBS-soluble tissue fraction was prepared from each mouse by homogenization with an Omni Bead Ruptor 24 (Omni International). Samples were homogenized in PBS lysis buffer containing 1 mM phenylmethylsulfonyl fluoride (Sigma #P7626), 0.5 mM EDTA, and 0.2X Halt Protease Inhibitor Cocktail (Thermo Scientific #87786) and centrifuged at 12,000 × *g* for 20 min at 4 °C. Supernatants were collected for Aβ_1–40_ (Aβ40)/Aβ_1–42_ (Aβ42) measurement using a human 6E10 Aβ kit (K15200E). All samples were run undiluted. Aβ peptide levels were normalized to the total mass of protein in the sample as determined by BCA Protein Assay (ThermoFisher #23225). Results were analyzed by using a general linear model with sex and treatment status as main effects.

## Results

**FIGURE 1:**
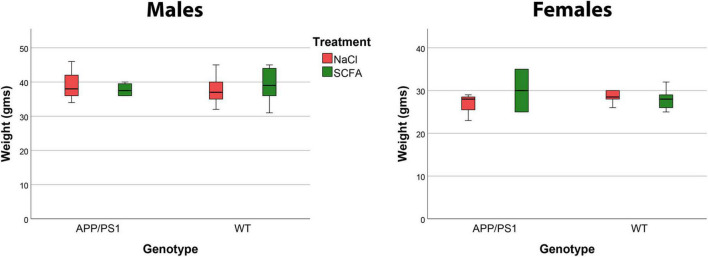
Mouse body weights were not affected by short chain fatty acid (SCFA) supplementation. Male mice weighed significantly more than female mice (*p* < 0.0001), but body weight was not influenced by SCFA-supplemented drinking water **(A)** or by the presence of the *APP/PS1* transgenes **(B)** (*p* > 0.05). These data reflect weights on the day of euthanasia.

The purpose of this study was to test the effects of SCFA supplementation on APP/PS1 mice maintained in a standard laboratory environment. The SCFA and saline control treatments were well-tolerated by the mice. Mice maintained healthy coats and body weights were unaffected (Figure 1).

**FIGURE 2:**
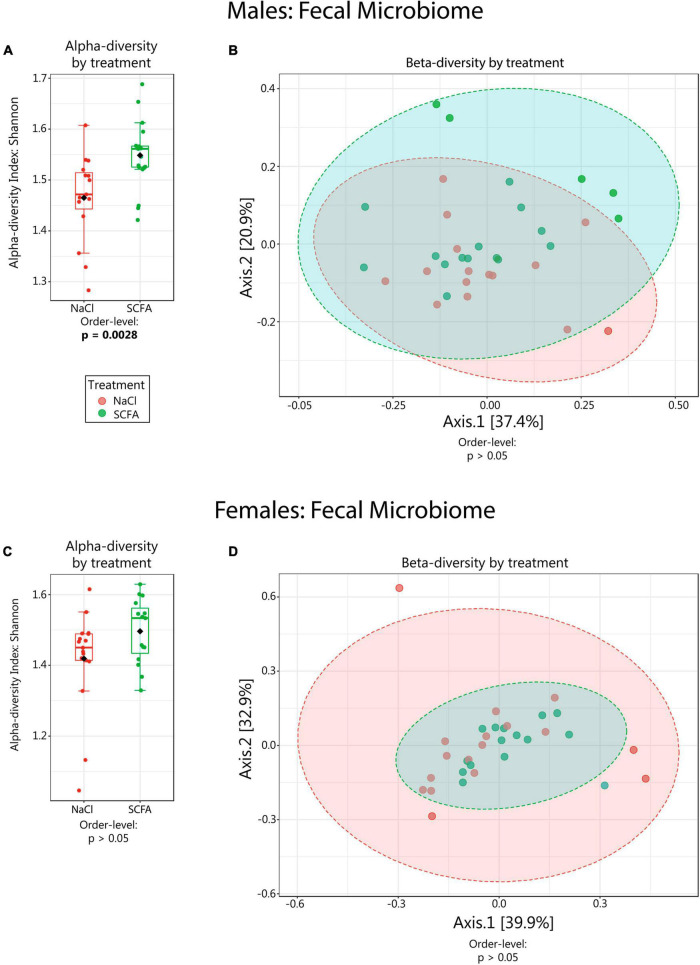
Microbiome alpha- and beta-diversity as a function of short chain fatty acid (SCFA) treatment. Alpha-diversity (Shannon H index) data are depicted in boxplots **(A,C)**. Beta-diversity analyses are visualized in PCoA plots **(B,D)**. Statistical significance for the findings is indicated below each graph. Ellipses in **(B,D)** represent 95% confidence intervals. For male mice **(B)** R^2^ = 0.035 and for female mice **(D)** R^2^ = 0.023. Beta-diversity was also analyzed using PERMDISP, which had no significant *p*-values except in females at the species level (*p* = 0.027), indicating that there was generally no difference in dispersion between SCFA vs saline treated groups.

The effects of SCFA supplementation on the gut microbiome have not been reported previously. Therefore, we analyzed 16S rRNA gene amplicon sequencing results from fecal DNA samples. Since sex impacts the amyloid burden in the APP/PS1 model, males and females were analyzed separately (Onos et al., 2019). We began with microbiome alpha-diversity (Shannon H index), which is a measure of within-sample diversity based on the richness and evenness of the taxa present. An alpha-diversity score was calculated using the Shannon H index for each sample. Both male and female mice showed a trend towards higher alpha-diversity with SCFA treatment, with male mice reaching statistical significance at the taxonomic levels of order (*p* = 0.0027), class (*p* = 0.0027) and phylum (*p* = 0.043) and female mice reaching significance at the genus (*p* = 0.049) taxonomic level (Figures 2A,C, *p*-values for all taxonomic levels in Table 1). The presence of the transgene had no significant effect on alpha diversity at any taxonomic level in males or females (*p* > 0.05). An increase in gut alpha-diversity, as associated with SCFA treatment here, is generally considered to be an indication of a healthier gut (Manor et al., 2020).

**TABLE 1:** Microbiome alpha-diversity was significantly associated with short chain fatty acid (SCFA) supplementation in male mice.

Alpha-diversity *p*-values		
	Males	Females
Species	0.17	0.43
Genus	0.056	**0.049**
Family	0.19	0.37
Order	**0.0027**	0.15
Class	**0.0027**	0.18
Phylum	**0.043**	0.29

*P-values reflect nominal p-values and were determined using Kruskal-Wallis tests. Values less then 0.05 are presented in bold font.*

Beta-diversity is a measure of similarity of the microbial communities between samples. Beta-diversity scores were visualized using PCoA plots with Bray-Curtis distance measures. In both male and female mice, beta-diversity was generally not significantly affected by SCFA treatment (Table 2). However, in male mice, beta-diversity was significantly associated with SCFA treatment on the genus level (R^2^ = 0.073, *p* < 0.049), while in female mice, it was significantly associated with SCFA treatment on the species level (R^2^ = 0.086, *p* < 0.009) (Figures 2B,D, and scores for all taxonomic levels in Table 2). Although the p-values are significant, the R^2^ values are low, a result of variability within group and overlap between groups, so these results may not have biological meaning.

**TABLE 2:** Microbiome beta-diversity did not significantly associate with short chain fatty acid (SCFA) treatment in both male and female mice.

Beta-Diversity scores			
Taxonomic level		Males	Females
Species	***p*-value**	0.300	**0.009**
	**R^2^**	0.036	0.086
Genus	***p*-value**	**0.049**	0.140
	**R^2^**	0.073	0.051
Family	***p*-value**	0.120	0.160
	**R^2^**	0.052	0.051
Order	***p*-value**	0.310	0.550
	**R^2^**	0.035	0.023
Class	***p*-value**	0.280	0.720
	**R^2^**	0.038	0.015
Phylum	***p*-value**	0.440	0.440
	**R^2^**	0.026	0.028

*The R^2^ values represent the proportion of the variance captured by SCFA vs saline treatment. The p-values were derived from analysis of 999 randomized permutations. P-values < 0.05 are presented in bold font.*

**FIGURE 3:**
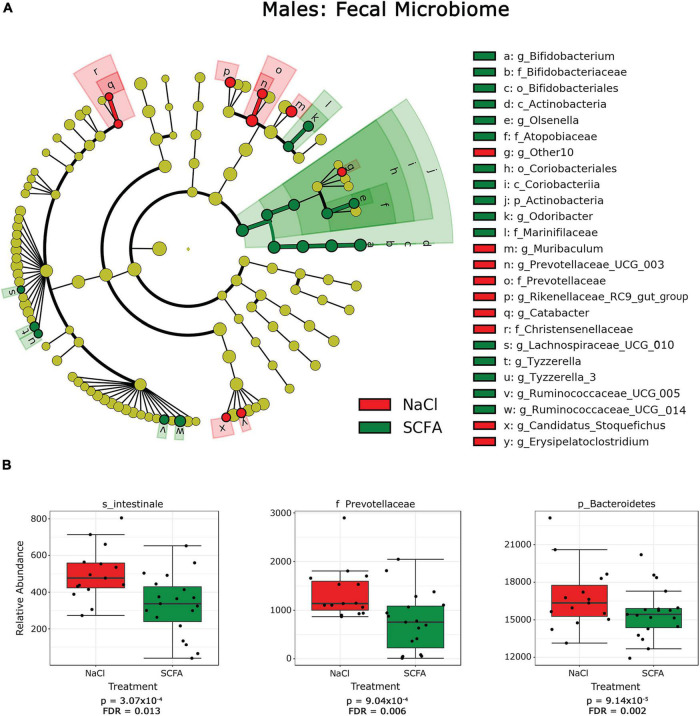
Cladograms and box plots of individual taxa reveal microbial phylogenetic branches associated with short chain fatty acid (SCFA) supplementation in male mice. **(A)** Taxa are represented as nodes that are connected by lines based on the phylogenetic relatedness of all taxa present in each experimental cohort. For example, the end node, a, represents the genus Bifodobacterium which is connected to other nodes representing higher level taxa related to Bifodobacterium including b the family Bifidobacteriaceae, c the order Bifidobacteriales, and d the class Actinobacteria. Many taxa are associated with SCFA vs saline treatment, with node colors indicating treatment with highest levels of each taxa. Statistical significance reflects both *p* < 0.05 for Kruskal-Wallis tests and a logarithmic LDA score > 2.0. **(B)** Box plots present the relative abundance of individual taxa that are significantly associated with SCFA supplementation by classical univariate analysis. A full list of taxa with nominal *p*-values and FDR corrected *p*-values is provided in Supplementary Material 1.1–1.6.

**FIGURE 4:**
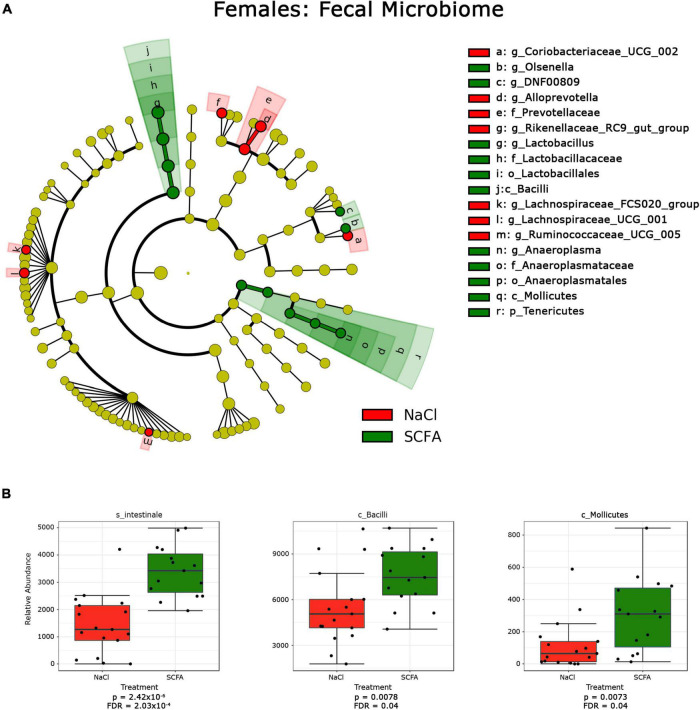
Cladograms and box plots of individual taxa reveal microbial phylogenetic branches associated with short chain fatty acid (SCFA) supplementation in female mice. **(A)** Statistical significance reflects both *p* < 0.05 for Kruskal-Wallis tests and a logarithmic LDA score > 2.0. **(B)** Box plots present the relative abundance of individual taxa that are significantly associated with SCFA supplementation by classical univariate analysis. A full list of taxa with nominal p-values and FDR corrected *p*-values is provided in Supplementary Material 1.1–1.6.

Since alpha- and beta-diversity measures suggest some significant SCFA effects on the gut microbiome, additional analyses to identify specific taxa were performed. Taxa significantly associated with SCFA treatment were visualized with cladograms portraying phylogenetic relatedness between the taxa (Figures 3A, 4A). Significance was calculated using a linear discriminant analysis of effect size (LefSe). An additional classical univariate analysis with FDR-corrected p-values was used to generate box plots of individual taxa significantly associated with SCFAs at all taxonomic levels (Figures 3B, 4B). Both analyses identified similar taxonomic trends, with the classical univariate analyses identifying additional taxa.

In male mice, SCFA supplementation resulted in a significant increase in the relative abundance of the phylum Actinobacterium, which includes the order Bifidobacteriales of the class Actinobacterium and the order Coriobacteriales of the class Coriobacteria (Figure 3A). In contrast, SCFA treatment resulted in a significant decrease in the families Prevotellaceae and Christensenellaceae and the genus *Olsenella* (Figure 3; Supplementary Material 1.1–1.6).

In female mice, SCFA supplementation significantly increased relative abundance of the genus *Anaeroplasma* of the phylum Tenericutes, and the genus *Lactobacillus* of the class Bacilli. In both sexes, the genus *Olsenella* was significantly increased in association with SCFA treatment, while the relative abundance of the family Prevotellaceae was decreased. Interestingly, SCFA treatment resulted in a significant increase of the species *Intestinale* from genus *Muribaculum* in female mice (Figure 4B), but a decrease in male mice (Figure 3B).

**FIGURE 5:**
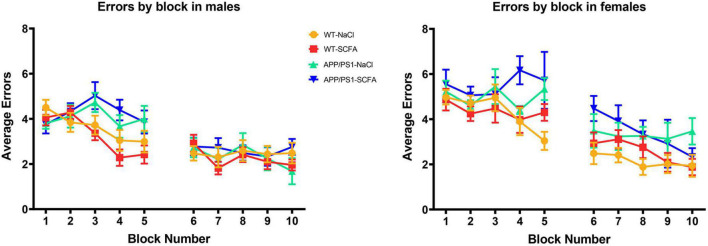
RAWM results for day one and day two trials. Data are represented as means of three trials per block of all animals within the group, with five blocks per day. Day one was training day, while day two was testing day. Error bars are standard deviation of the mean.

**FIGURE 6:**
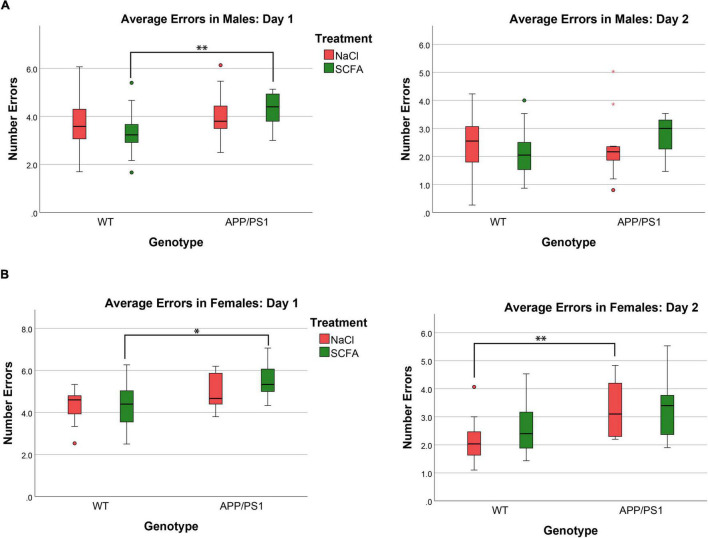
Female but not male mice show cognitive deficits that are unaffected by short chain fatty acids (SCFA) treatment. The average number of errors on day one and day two are shown. On day one, significant differences were observed for both male and female mice between SCFA treated WT vs. APP/PS1 mice **(A,B)**. On day two, significant differences were observed only for female saline-treated WT vs. APP/PS1 mice **(B)**. Statistical significance was determined by using a general linear model (* = *p* < 0.05, ^**^ = *p* < 0.01).

To determine whether SCFA supplementation improved cognition in the APP/PS1 mice, the animals were subjected to a RAWM test. The number of errors observed in male and female mice was determined on day one and day two (Figure 5). Since larger amyloid burden has been observed in female mice in this model (Onos et al., 2019), results from male and female mice were analyzed separately. These results were analyzed by a general linear model that included treatment status, transgene status, and treatment-transgene interaction as main effects (Figure 6).

The overall statistical model for male mice on day one was statistically significant (F_3_,_60_ = 3.674, *p* = 0.017), where transgene was significant (*p* = 0.005), but treatment and treatment-transgene interaction were not significant [(*p* = 0.789) and (*p* = 0.264), respectively]. Regarding transgene effects, the SCFA-treated APP/PS1 mice made more errors than the SCFA-treated WT mice (*p* = 0.004, Figure 5). However, the number of errors committed by the saline-treated APP/PS1 mice was not significantly different from saline-treated WT mice (*p* = 0.218). Overall, these findings indicate no treatment effect, and a transgene effect only in SCFA-treated mice.

The overall statistical model for female mice on day one was statistically significant (F_3_,_39_ = 3.077, *p* = 0.039), where transgene was significant (*p* = 0.005), but treatment and treatment-transgene interaction were not significant [(*p* = 0.454) and (*p* = 0.351), respectively]. Regarding transgene effects, SCFA-treated APP/PS1 mice made more errors than the SCFA-treated WT mice (*p* = 0.016, Figure 5). In contrast, saline-treated APP/PS1 mice were not significantly different from saline-treated WT mice (*p* = 0.103). Overall, these findings again indicate no treatment effect in either APP/PS1 or WT mice and a transgene effect only in SCFA-treated mice.

The overall statistical model for males on day two was not statistically significant (F_3_,_58_ = 1.068, *p* = 0.370), where the overall transgene effect, treatment, and treatment-transgene interaction were not significant (*p* = 0.334, *p* = 0.816, and *p* = 0.153, respectively, Figure 6). Overall, male mice did not display any significant difference in errors made on day two based on treatment, transgene, or a combined treatment-transgene interaction.

The overall statistical model for females on day two was statistically significant (F_3_,_39_ = 3.746, *p* = 0.019), where the overall transgene effect was significant (*p* = 0.003), while the treatment and treatment-transgene interaction were not significant (*p* = 0.445 and *p* = 0.606, respectively). On day two, the female saline-treated APP/PS1 mice made more errors than the saline-treated WT mice (*p* = 0.008, Figure 6). The female SCFA-treated APP/PS1 mice showed a similar trend towards more errors than the SCFA-treated WT mice, but this trend did not reach significance (*p* = 0.08, Figure 6). In summation, the female mice showed a robust transgene effect, especially in the saline-treated mice, while treatment and a treatment-transgene interaction were not significant.

**FIGURE 7:**
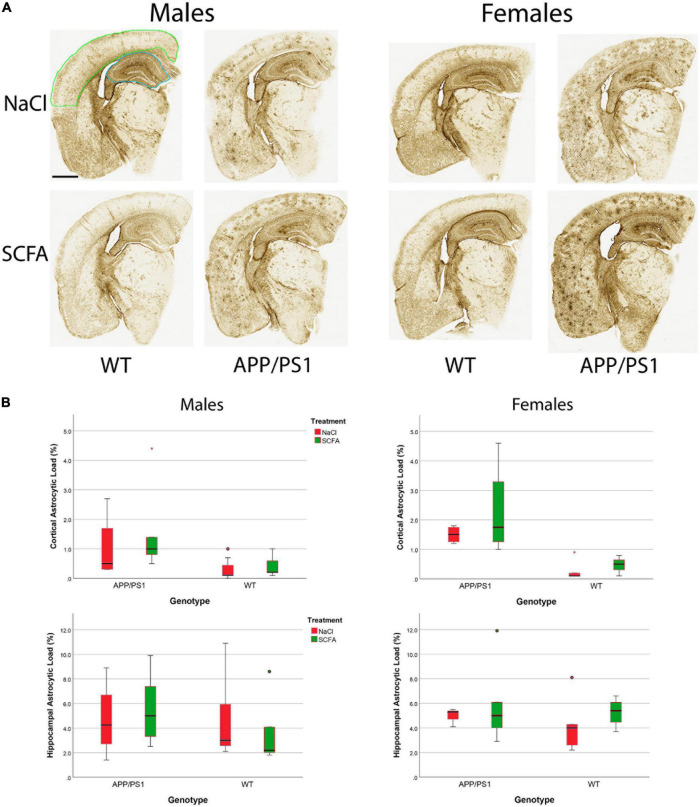
Gfap staining and quantification indicate short chain fatty acids (SCFA) has no effect although labeling is increased in cortices of APP/PS1 mice. Gfap expression was detected by Gfap immunohistochemistry **(A)**. Scale bar represents 1 mm. Green outlining indicates the typical ROI for cortical staining while blue outlining indicates the ROI for hippocampal staining. Statistical analysis confirms a significant difference with APP/PS1 transgenes but not SCFA treatment in the cortex **(B)**. Staining was quantified using HALO Area Quantification v.2.2.1. Statistical significance was determined by using a general linear model. Datapoints marked as circles represent ourliers (3rd quartile + 1.5 × interquartile range) while asterisks mark extreme outliers (3rd quartile + 3 × interquartile range).

To determine SCFA effects on glial activation in the mice, Gfap immunohistochemistry was performed (Figure 7). HALO was used to quantify the extent of robust Gfap staining in cortical and hippocampal slices. Results are presented as the percent of the region of the interest that was strongly Gfap positive (Figure 7). As detailed below, SCFA treatment did not significantly affect staining in the cortex or hippocampus in either male or female mice. The presence of the APP/PS1 transgenes significantly increased Gfap staining only in the cortex (Figure 7).

The overall statistical model for cortical Gfap staining in male mice was significant (F_3_,_17_ = 3.554, *p* = 0.037), where the transgene effect was significant (*p* = 0.008), the treatment effect was not significant (*p* = 0.333), and the transgene-treatment interaction was not significant (*p* = 0.622, Figure 7). Specifically, the presence of the APP/PS1 transgenes significantly increased Gfap in the SCFA-treated mice (*p* = 0.020), while the saline-treated mice showed a similar trend (*p* = 0.106).

The overall statistical model for cortical Gfap staining in female mice was also significant (F_3_,_12_ = 9.205, *p* = 0.002), where the transgene effect was significant (*p* < 0.001), and neither the treatment effect nor the transgene-treatment interaction were significant (*p* = 0.273 and *p* = 0.637, respectively). Specifically, the presence of the APP/PS1 transgenes significantly increased Gfap in both the SCFA-treated mice (*p* = 0.014) and the saline-treated mice (*p* = 0.002).

The overall statistical models for hippocampal Gfap staining in male mice and female mice were not significant [(F_3_,_17_ = 0.443, *p* = 0.0726) and (F_3_,_14_ = 0.680, *p* = 0.579), respectively]. For each model, transgene, treatment, and transgene-treatment interaction were not significant.

**FIGURE 8:**
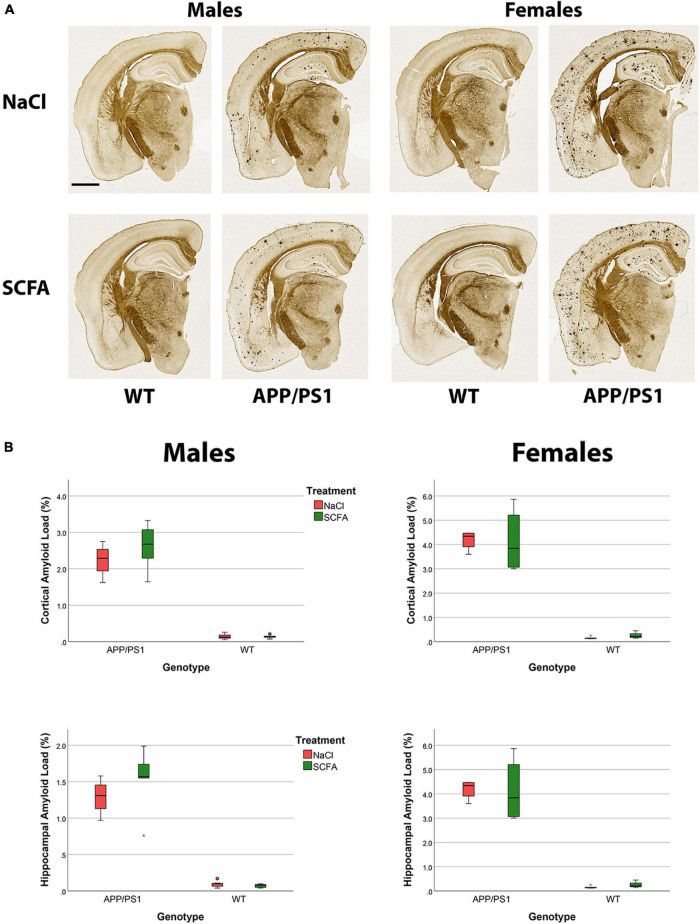
Amyloid staining and quantification indicate no significant short chain fatty acids (SCFA) effect although an increased amyloid load is observed in female mice. The Campbell–Switzer silver stain labels both parenchymal amyloid and cerebral vascular amyloid **(A)**. Scale bar represents 1 mm. Statistical analysis confirms a significant difference with sex and genotype but not SCFA treatment **(B)**. Staining was quantified using HALO Area Quantification v.2.2.1. Statistical significance was determined by using a general linear model. Datapoints marked as circles represent ourliers (3rd quartile +/− 1.5 × interquartile range) while asterisks mark extreme outliers (3rd quartile +/− 3 × interquartile range).

To determine SCFA effects on amyloid accumulation in the APP/PS1 mice, amyloid burden was quantified in the cortex and in the hippocampus by both histochemistry and by MSD. For the histochemistry, HALO was used to quantify the percent area of the cortex and hippocampus ROI that was amyloid positive, and results analyzed by using a general linear model (Figure 8). The overall statistical models for cortical and hippocampal amyloid load were statistically significant [(F_3_,_33_ = 69.199, *p* < 0.001) and (F_3_,_33_ = 68.610, *p* < 0.001), respectively]. In the cortex, SCFA treatment again had no significant effect on amyloid (F_1_,_33_ = 0.174, *p* = 0.679). In the hippocampus, SCFA treatment had no significant effect (F_1_,_33_ = 0.071, *p* = 0.791) on amyloid burden.

Independent of treatment, female mice appeared to have robust amyloid deposition while male mice appeared to have a lighter amyloid burden (Figure 8A), as has been reported previously for this murine model (Onos et al., 2019). Statistical analysis of the amyloid staining confirmed that female mice had a greater amyloid burden than male mice in the cortex (F_1_,_33_ = 14.126, *p* < 0.001) and in the hippocampus (F_1_,_33_ = 14.484, *p* < 0.001, Figure 8B).

**FIGURE 9:**
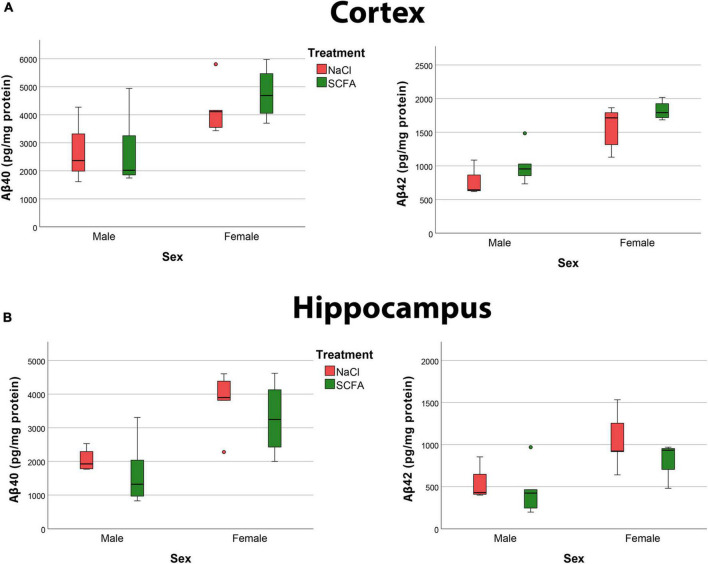
Amyloid MSD indicates no significant effect of short chain fatty acids (SCFA) on Aβ40 and Aβ42 and confirm that female mice had significantly more Aβ. Quantification of Aβ40 and Aβ42 in the cortex **(A)** and hippocampus **(B)** reveals a significant difference with sex but not with SCFA treatment. Statistical significance was determined by using a general linear model.

To discern Aβ40 and Aβ42 independently, soluble amyloid peptide levels were quantified by MSD analyses. The results revealed robust levels of Aβ40 and Aβ42 in both the cortex and hippocampus (Figure 9). The overall models for cortical Aβ40 (F_2_,_15_ = 5.360, *p* = 0.018), hippocampal Aβ40 (F_2_,_15_ = 9.146, *p* = 0.003), cortical Aβ42 (F_2_,_15_ = 21.492, *p* < 0.001), and hippocampal Aβ42 (F_2_,_15_ = 6.712, *p* = 0.008), were statistically significant.

Short chain fatty acid treatment did not significantly impact cortical Aβ40 (F_1_,_15_ = 0.375, *p* = 0.549), hippocampal Aβ40 (F_1_,_15_ = 1.069, *p* = 0.318), cortical Aβ42 (F_1_,_15_ = 3.549, *p* = 0.079), or hippocampal Aβ42 (F_1_,_15_ = 1.204, *p* = 0.290).

Independent of treatment, female mice had significantly more cortical Aβ40 (F_1_,_15_ = 10.705, *p* = 0.005), hippocampal Aβ40 (F_1_,_15_ = 16.077, *p* = 0.001), cortical Aβ42 (F_1_,_15_ = 42.798, *p* < 0.001), and hippocampal Aβ42 (F_1_,_15_ = 11.238, *p* = 0.004) compared to the male mice (Figure 9). Overall, these findings that female mice had significantly more amyloid than male mice, but that SCFA treatment had no significant effect on amyloid, confirm the results of the histochemistry analyses.

## Discussion

The goal of this study was to test the effects of SCFA supplementation in APP/PS1 mice maintained in a conventional laboratory animal environment. Our primary findings were that SCFA treatment significantly impacted the gut microbiome but had no effect on spatial memory deficits, glial activation nor amyloid burden in this model. In both male and female mice, SCFA supplementation was associated with increased alpha-diversity and increased relative abundance of several taxa associated with SCFA-production. Although a behavioral deficit was associated with the APP/PS1 transgenes, no effects were detected following SCFA treatment. SCFA supplementation also had no significant effect on Gfap expression or amyloid burden. Overall, we interpret these findings as indicating that SCFA supplementation affects the gut microbiome, but not the hallmarks associated with this preclinical model of AD.

Short chain fatty acid supplementation increased several bacteria in male and female mice. Based on LefSe analyses, the genera *Bifidobacterium, Olsenella* and *Odoribacter* increased in male mice, and the genera *Lactobacillus, Olsenella* and *Anaeroplasma* increased in female mice. Interestingly, of these taxa, the genera *Bifidobacterium* and *Lactobacillus* are known to produce SCFAs and SCFA precursors [reviewed in Markowiak-Kopec and Slizewska (2020)]. Overall, these findings suggest a possible feedforward effect of SCFA supplementation, in which SCFAs increase the relative abundance of bacteria that produce SCFAs. Further studies are needed to corroborate this finding.

Several studies report a role of SCFAs in the brain. For example, treatment of astrocytes or microglia *in vitro* with the SCFA acetate has been shown to reverse LPS-induced astrocytic activation and inhibit NFkB signaling (Soliman et al., 2012, 2013). *In vivo*, SCFA treatment impacts microglial morphology, transcriptome, and response to stimuli, such as LPS (Erny et al., 2015). The mechanisms by which SCFAs act on cells within the brain is under intense scrutiny. SCFAs have been found to inhibit histone deacetylation, thereby affecting gene expression and inflammation [reviewed in Silva et al. (2020)]. For example, treatment of mice with sodium butyrate reduces histone deacetylase (HDAC) activity in the gut, associated immune cells and the central nervous system (Reddy et al., 2018); [reviewed in Silva et al. (2020)]. A second mechanism that may mediate SCFA activity in the brain is the binding of SCFAs to free fatty acid receptors FFAR2 and FFAR3 [reviewed in Silva et al. (2020); Dalile et al. (2019)]. While these receptors are mostly expressed in intestinal mucosal cells and immune cells, FFAR3 is also expressed by neurons in the periphery (Bolognini et al., 2016). FFAR2 and FFAR3 expression in the brain has not been reported. SCFA actions in the brain depend upon their transport across the blood brain barrier. This is mediated by monocarboxylate transporters which are expressed at high levels in the endothelial cells of the blood brain barrier [reviewed in Silva et al. (2020)].

Several factors may influence SCFA levels *in vivo*. First, diet has been shown to clearly modulate SCFA levels because components of soluble fiber such as inulin are metabolized to SCFAs by the gut microbiome [reviewed in den Besten et al. (2013); Pellizzon (2016)]. In this study, the mice were maintained on the Teklad Global 18% Protein Rodent Diet which contains ingredients such as ground wheat, ground corn and wheat middlings that are a source of soluble fiber that is metabolized to SCFAs by the gut (den Besten et al., 2013; Pellizzon, 2016; Envigo, 2022). Second, the profile of bacteria within the gut impacts SCFA levels because certain bacteria are particularly proficient at generating SCFAs (Vital et al., 2014), [reviewed in Markowiak-Kopec and Slizewska (2020)]. Third, genetics may impact SCFA levels. For example, *APOE4*, which is associated with increased LDL-cholesterol and AD risk, relative to *APOE3*, is associated with a gut microbiome profile with reduced SCFA-producing bacteria, such as Ruminococcaceae (Maldonado Weng et al., 2019; Tran et al., 2019; Parikh et al., 2020; Zajac et al., 2022).

Because the gut microbiome produces SCFAs and has been shown to modulate AD pathology, and SCFAs may act in the brain, several groups have investigated the effects of SCFA treatment on amyloid accumulation in the brain. Results have not been consistent (Table 3). Colombo et al. found that SCFA treatment increased amyloid burden in APPPS1 mice (Colombo et al., 2021). In contrast, Fernando et al. and Jiang et al. found that sodium butyrate treatment reduced amyloid burden in 5xFAD mice (Fernando et al., 2020; Jiang et al., 2021). Here, we treated APP/PS1 mice with SCFAs and found no effect on amyloid burden. In the following paragraphs, we will compare and contrast prior results with the results presented here.

**TABLE 3:** Comparison of studies evaluating short chain fatty acids (SCFA) effects on murine amyloid models.

Study	Treatment	Mouse model	Microbiome	Mouse age during treatment	Effect on amyloid burden
Fernando et al., 2020	NaB was added to chow pellets at a concentration of either 40 mg/kg or 120 mg/kg where mice would receive either 5 mg/kg/day, or 15 mg/kg/day	5xFAD with APOE3	Conventional	From 8 weeks to 20 weeks	Reduced
Jiang et al., 2021	0.2 g/kg daily intraperitoneal injection of NaB (0.1 ml/10 g) *	5xFAD	SPF	From 8 weeks to 10 weeks	Reduced
Colombo et al., 2021	67.5 mM sodium acetate, 25 mM sodium propionate, 40 mM sodium butyrate, pH 6.8 in drinking water	APPPS1	SPF	From 8 weeks to 13 weeks	Increased
Zajac et al., 2022	67.5 mM sodium acetate, 25 mM sodium propionate, 40 mM sodium butyrate, pH 6.8 in drinking water *	APP/PS1	Conventional	From 20 weeks to 40 weeks	No change

*SCFA treatment was compared to saline control.

Differences in the experimental designs of these studies are multiple (Table 3). One difference is the mode of SCFA administration and type of SCFA. Our study and that of Colombo et al. were similar in that SCFAs were administered in the drinking water at identical concentrations. However, they differ in that the control group in Colombo et al. received water while the control group in our study received saline (132.5mM) as their drinking water such that their sodium intake was equal to that of the SCFA treated mice. Interestingly, APP/PS1 mice that received about three-times more sodium chloride than our control group were reported to have reduced amyloid plaques (Bachmanov et al., 2002; Taheri et al., 2016). Whether comparing SCFA treatment to saline treatment may have obscured a SCFA effect in our study is not clear. Jiang et al. used intraperitoneal injection of sodium butyrate while Fernando et al. administered sodium butyrate *via* chow (Fernando et al., 2020; Jiang et al., 2021).

These SCFA studies also used different mouse models (Table 3). The Colombo et al. and Jiang et al. studies used mice maintained on a SPF microbiome background while mice in our study and Fernando et al. were maintained with a conventional microbiome. Since altering the microbiome with antibiotics reduces amyloid burden, differences in the microbiome may contribute to the differences observed in these studies (Minter et al., 2016, 2017; Guilherme et al., 2021). The mouse models also differed in that Colombo et al. used an APPPS1 model wherein the *APPPS1* transgenes were driven by the Thy1 promoter, and the PS1 mutation was L166P (Colombo et al., 2021). This mouse model begins to deposit amyloid at six weeks of age and Columbo et al. started SCFA treatment at eight weeks of age for a duration of five weeks. Jiang et al. and Fernando et al. used the 5xFAD murine model wherein extracellular amyloid deposits begin at eight weeks of age. Both studies began butyrate treatment at eight weeks. Jiang et al. treated for two weeks while Fernando et al. treated for 12 weeks. In our study, the *APP/PS1* transgene was driven by the mouse prion protein promoter, and the PS1 mutation was deletion of exon 9 (Jankowsky et al., 2004). This mouse model begins to deposit amyloid at four to six months of age. We began SCFA treatment at five months of age for a duration of five months. Hence, the studies are similar in that mice underwent SCFA treatment during the time that amyloid was accumulating. The studies are different in that (i) Colombo et al., Jiang et al. and Fernando et al. used mouse models with earlier amyloid deposition compared to the APP/PS1 model in our study; and (ii) Colombo et al. and Jiang et al. used SPF mice while Fernando et al. and our study used mice with a conventional microbiome.

Considering these variables and the mixed study results, we speculate the treatment with butyrate *per se* reduces amyloid burden because similar results were found with two different routes of administration and with SPF and conventional microbiomes (Table 3). In contrast, treatment with a mixture of acetate, propionate and butyrate produces results that appear model dependent. We propose that future studies investigating the effects of individual SCFAs may provide clarity to this field.

## Conclusion

This is the first study to robustly evaluate SCFA supplementation effects on the gut microbiome itself, in addition to brain pathology and behavior, which have been reported on in previous studies. We found that SCFA treatment increased levels of SCFA-producing bacteria *Lactobacillus* and *Bifidobacterium* in a possible feedforward mechanism. Consistent with prior reports, female APPswe/PSEN1dE9 mice had a greater amyloid burden and memory deficit than male mice (Onos et al., 2019). However, inconsistent with prior reports, we did not detect an effect of SCFA supplementation on behavioral impairment or amyloid burden. We recognize that murine models of AD are pre-clinical, and so results are used to inform more physiologically relevant human studies. Given the conflicting results in these pre-clinical models, further studies are necessary to provide clarity to this emerging area.

## Data Availability Statement

The datasets presented in this study can be found in online repositories. The names of the repository/repositories and accession number(s) can be found below: Raw sequence data files were submitted to the Sequence Read Archive (SRA) of the National Center for Biotechnology Information (NCBI) under the BioProject Identifier: PRJNA809693.

## Ethics Statement

The animal study was reviewed and approved by University of Kentucky Institutional Animal Care and Use Committee.

## Author Contributions

DZ, DB, SG, JM, and SE contributed to conception and design of the study. DZ and SG organized the database. DZ, BS, and SE performed the statistical analysis. DZ wrote the first draft of the manuscript. All authors contributed to manuscript revision, read, and approved the submitted version.

## Conflict of Interest

The authors declare that the research was conducted in the absence of any commercial or financial relationships that could be construed as a potential conflict of interest.

## Publisher’s Note

All claims expressed in this article are solely those of the authors and do not necessarily represent those of their affiliated organizations, or those of the publisher, the editors and the reviewers. Any product that may be evaluated in this article, or claim that may be made by its manufacturer, is not guaranteed or endorsed by the publisher.
