# Supplementary figures and images for: Exogenous Short Chain Fatty Acid Effects in APP/PS1 Mice
Source: Front Neurosci. 2022 Jul 4;16:873549. doi: 10.3389/fnins.2022.873549 (PMC9289923; doi:10.3389/fnins.2022.873549)

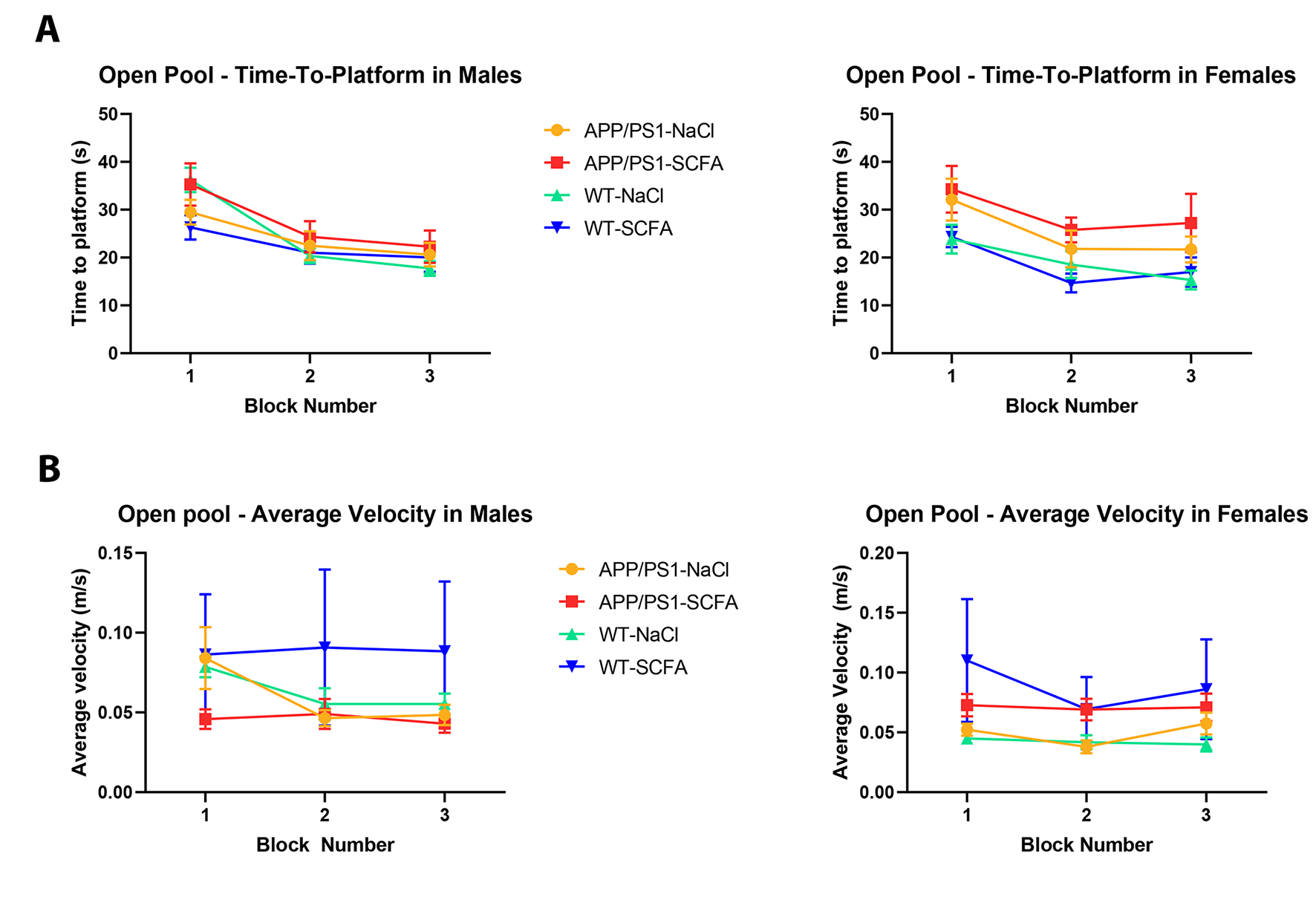

Supplement: Supplementary file 1 [file Image_1.TIFF]
